# A prospective interventional study evaluating seizure activity during a radiotherapy course for high-grade gliomas (SURF-ROGG)

**DOI:** 10.1186/s12885-021-08121-y

**Published:** 2021-04-09

**Authors:** Dirk Rades, Jaspar Witteler, Denise Olbrich, Peter Trillenberg, Steven E. Schild, Soeren Tvilsted, Troels W. Kjaer

**Affiliations:** 1 Department of Radiation Oncology, University of Lübeck, Ratzeburger Allee 160, 23562 Lübeck, Germany; 2 The Centre for Clinical Trials Lübeck, Lübeck, Germany; 3 Department of Neurology, University of Lübeck, Lübeck, Germany; 4 Department of Radiation Oncology, Mayo Clinic Scottsdale, Scottsdale, AZ USA; 5 Research Projects and Clinical Optimization, Zealand University Hospital, Koege, Denmark; 6 Neurological Department, Zealand University Hospital, Roskilde, Denmark

**Keywords:** High-grade glioma, Seizures, Radiotherapy, Radio-chemotherapy, Seizure diary

## Abstract

**Background:**

Gliomas are often associated with symptoms including seizures. Most patients with high-grade gliomas are treated with radiotherapy or radio-chemotherapy. Since irradiation causes inflammation, it may initially aggravate symptoms. Studies focusing on seizure activity during radiotherapy for gliomas are not available. Such knowledge may improve patient monitoring and anti-epileptic treatment. This study evaluates seizure activity during radiotherapy for high-grade gliomas.

**Methods:**

The primary objective this prospective interventional study is the evaluation of seizure activity during a course of radiotherapy for high-grade gliomas. Progression of seizure activity is defined as increased frequency of seizures by > 50%, increased severity of seizures, or initiation/increase by ≥25% of anti-epileptic medication. Seizure frequency up to 6 weeks following radiotherapy and electroencephalography activity typical for epilepsy will also be evaluated. Patients keep a seizure diary during and up to 6 weeks following radiotherapy. Every day, they will document number (and type) of seizures and anti-epileptic medication. Once a week, the findings of the diary are checked and discussed with a neurologist to initiate or adjust anti-epileptic medication, if necessary. Patients complete a questionnaire regarding their satisfaction with the seizure diary. If the dissatisfaction rate is > 40%, the seizure diary will be considered not suitable for the investigated indication. Thirty-five patients (32 patients plus drop-outs) should be enrolled. With this sample size, a one-sample binomial test with a one-sided significance level of 2.5% has a power of 80% to yield statistical significance, if the rate of patients with progression of seizure activity is 30% (rate under the alternative hypothesis), assuming a ‘natural’ background progression-rate of 10% without radiotherapy (null hypothesis).

**Discussion:**

If an increase in seizure activity during a course of radiotherapy for high-grade glioma occurs, the findings of this study may pave the way for a larger prospective trial and will likely lead to closer patient monitoring and better anti-epileptic treatment.

**Trial registration:**

clinicaltrials.gov (NCT04552756); registered on 16th of September, 2020.

## Background

Gliomas represent the most common type of primary brain tumors and are frequently associated with clinical symptoms including seizures [1–7]. The majority of patients with high-grade gliomas (grade III or IV according to the classification of the World Health Organization) receive radiotherapy with or without chemotherapy, either as adjuvant treatment after neurosurgical resection or as definitive treatment after biopsy. Due to an acute inflammatory reaction associated with edema in the irradiated area of the brain and subsequent increase of intracranial pressure, radiotherapy may lead to onset or progression of clinical symptoms including seizures during the course of treatment [8, 9]. To our knowledge, no studies are available that focused on the subacute effect of radiotherapy on seizure activity during a course of radiation treatment in glioma patients. These data would be important to improve monitoring and anti-epileptic treatment of these patients. This study evaluates the seizure activity during radiotherapy for high-grade (grade III or IV) gliomas.

## Methods and design

This single-arm prospective interventional study performed at a single academic center will evaluate the seizure activity during radiotherapy of high-grade gliomas. The study has been approved by the local ethics committee (University of Lübeck, no. 20–311) and registered at clinicaltrials.gov (identifier: NCT04552756).

### Objectives and endpoints

Primary objective is the evaluation of seizure activity during a course of radiotherapy for high-grade gliomas. Progression of seizure activity during the course of radiotherapy compared to baseline is defined as increase of frequency of seizures by more than 50%, increase of severity of seizures (i.e. increase of generalized seizures by more than 50%), or increase of the dose of anti-epileptic medication by at least 25% or initiation of anti-epileptic medication. The latter criterion was defined in accordance with another situation in radiation oncology, radiotherapy of painful bone metastases [10]. These parameters will be assessed using a seizure diary kept by the patients during the course of radiotherapy. In addition, seizure frequency up to 6 weeks following radiotherapy (seizure diary), patient satisfaction with the seizure diary, and electroencephalography (EEG) activity typical for epilepsy will be evaluated.

### Eligibility criteria

Inclusion criteria include histologically proven newly diagnosed or recurrent grade III or IV glioma (associated or not associated with seizures), indication for normo-fractionated radiotherapy, Eastern Cooperative Oncology Group performance score 0–2, age ≥ 18 years, written informed consent (taken by physicians registered as investigators for this trial), and capacity of the patient to cooperate (including the use of a seizure diary). Exclusion criteria include pregnancy/lactation and limited legal capacity or being under legal supervision.

### Assessments

Parameters assessed prior radiotherapy include demographics (age, date of birth, gender), medical history, concomitant diseases, concomitant medication including anti-epileptic treatment, physical examination, histology and grade of glioma, upfront surgery, and planned radiotherapy/radio-chemotherapy.

**Fig. 1:**
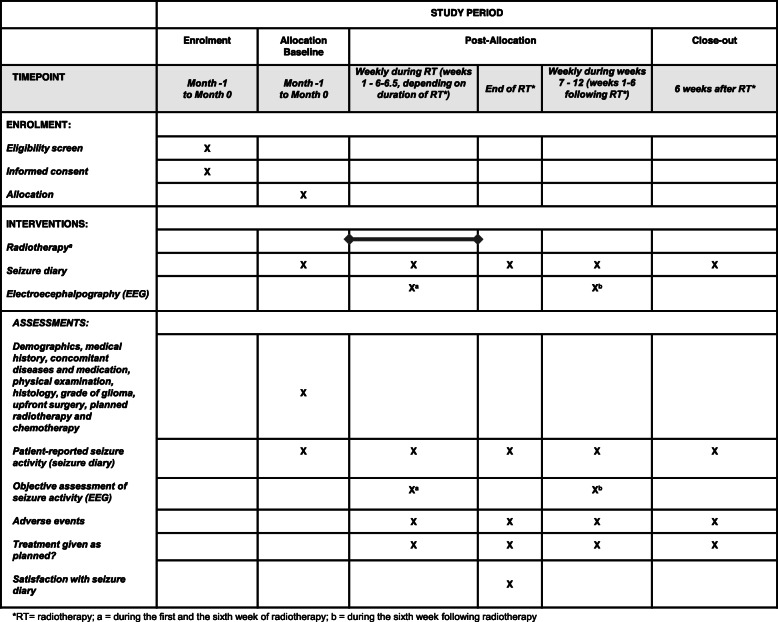
Timeline of enrolment, interventions and assessments of the SURF-ROGG trial

The following parameters will be assessed during the trial (Fig. 1):

1. Seizure frequency

The patients keep a seizure diary during radiotherapy and up to 6 weeks following radiotherapy. Every day, the patients document the number and type of seizures and intake of anti-epileptic medication. At the end of radiotherapy, the patients are asked to complete a questionnaire regarding their satisfaction with the seizure diary.

2. Objective assessment of seizure activity

To obtain an objective assessment of seizure activity in addition to patient reported outcomes, an EEG is performed during the first and sixth week of radiotherapy, and during the sixth week following radiotherapy.

3. Adverse Events

Adverse events other than seizures will be assessed on an ongoing basis according to CTCAE v5.0 [11].

In addition, potential prognostic factors regarding the increase of seizure activity during the course of radiotherapy will be evaluated including seizure activity at baseline, age, gender, glioma grade, recurrent glioma, isocitrate dehydrogenase (IDH1/2) mutation status, methylation of the O^6^-methylguanine-DNA methyl-transferase (MGMT) gene promoter, tumor site, tumor size, cortical involvement and extent of upfront surgery.

## Interventions

Patients receive the same standard treatment for high-grade glioma as they would have received without participation in this study. If necessary, any care and interventions are permitted during the trial for treatment-related events and other types of comorbidity.

### Glioblastoma (grade IV)

The treatment of glioblastomas includes resection or biopsy of the tumor followed by radiotherapy of the tumor region plus margins. Radiotherapy is performed with a high-precision technique, i.e. volumetric modulated arc therapy (VMAT). Glioblastoma patients with a Karnofsky performance score ≥ 70 and younger than 70 years generally receive normo-fractionated radiotherapy [12]. This applies also to patients aged ≥70 years with methylation of the MGMT gene promoter [12]. The dose-fractionation regimen depends on the proximity of the tumor to structures and organs at risk and is either 30 × 2.0 Gy over 6 weeks or 33 × 1.8 Gy over 6.5 weeks. Tolerance doses of the structures and organs at risk will be considered using the Quantitative Analyses of Normal Tissue Effects in the Clinic (QUANTEC) [13].

Radiotherapy should be combined with temozolomide (TMZ), whenever reasonable. TMZ is given concurrently with radiotherapy plus sequentially following radiotherapy. The dose of concurrent TMZ is 75 mg/m2/day, administered on 7 days per week during the entire course of radiotherapy. Concurrent treatment is followed by 6 courses of sequential TMZ (150–200 mg/m2/day on 5 consecutive days every 4 weeks).

### Anaplastic astrocytoma and anaplastic oligodendroglioma (grade III)

The treatment of grade III gliomas also includes resection or biopsy of the tumor. Postoperative treatment varies between anaplastic astrocytomas and anaplastic oligodendrogliomas. Most patients receive postoperative radiotherapy. Anaplastic astrocytomas with IDH1/2 wild type are generally treated like glioblastomas. If anaplastic astrocytomas show an IDH1/2 mutation, postoperative treatment starts with radiotherapy alone followed by sequential chemotherapy with TMZ (150–200 mg/m2/day on 5 consecutive days every 4 weeks) or 4 courses of PCV including procarbazine (60 mg/m2 on days 8–21), lomustine (110 mg/m2/day on day 1) and vincristine (1.4 mg/m2/day, maximum absolute dose = 2.0 mg, on days 8 and 29, every (6-)8 weeks) [12]. To reduce toxicity, vincristine may be omitted. In case of an IDH1 mutation, astrocytomas and oligodendrogliomas can be differentiated by the 1p/19q co-deletion. Astrocytomas do not have a 1p/19q co-deletion, whereas the co-deletion is present in oligodendrogliomas. Postoperative treatment of anaplastic oligodendrogliomas with IDH mutation and 1p/19q co-deletion starts with radiotherapy alone followed by chemotherapy with 4 courses of PCV [12]. Again, vincristine may be omitted to reduce treatment-related toxicity.

### Seizure diary

The patients keep a seizure diary during the period of radiotherapy and up to 6 weeks following radiotherapy. Every day, the patients document the number and type of seizures and intake of anti-epileptic medication.

Once a week during the radiotherapy course, the seizure diary will be reviewed by a medical staff member. During the 6 weeks following radiotherapy, the patients are contacted by phone (to minimize the number of visits to the hospital) once a week to obtain the information from the seizure diary. During and following radiotherapy, the weekly findings of the seizure diary are discussed with a neurologist to initiate or adjust anti-epileptic medication, if necessary. At the end of radiotherapy, the patients are asked to complete a questionnaire regarding their satisfaction with the seizure diary. In case of a dissatisfaction rate > 40%, the seizure diary will be considered not suitable for patients with high-grade gliomas.

### Electroencephalography (EEG)

The patients receive an EEG during the first and the sixth week of radiotherapy, as well as during the sixth week following radiotherapy. Activity typical for epilepsy includes spike waves, sharp waves and/or sharp slow waves and is classified as absent or present.

## Sample size calculations

The main goal of the study is to generate objective data on the occurrence, frequency and severity of seizures and the use of anti-epileptic medication during the course of radiotherapy applying standardized questionnaires, in order to evaluate the potential effect of radiotherapy in patients with high-grade gliomas and generate hypotheses. Thirty-two patients are required for the statistical analyses. Assuming that 10% of patients do not fulfil these requirements, a total of 35 patients should be enrolled to this trial. With this sample size, a one-sample binomial test with a one-sided significance level of 2.5% has a power of 80% to yield statistical significance if the rate of patients with progression of seizure events during the course of radiotherapy is 30% (rate under the alternative hypothesis), assuming a ‘natural’ background progression-rate of 10% without radiotherapy (null hypothesis). The latter rate was chosen after discussions with experienced neurologists. If the natural course of the disease would lead to a progression-rate of 5% without radiotherapy, the power increases to 98%. Recruitment should be completed within 12 months. The radiotherapy period is 6–6.5 weeks, and follow up is 6 weeks. Thus, duration of the trial is about 15 months.

## Statistical methods for investigated endpoints

The focus of the statistical analysis is descriptive and exploratory in nature. If statistical tests are applied, they are to be interpreted on an exploratory perspective. All data recorded in the case report forms describing the study population (demographic and clinical characteristics, at baseline) will be analyzed descriptively. Categorical data will be presented in tables with frequencies and percentages. Continuous data will be summarized with at least the following: Frequency (n), median, quartiles, mean, standard deviation (standard error), minimum and maximum. Number of patients with protocol deviations during the study and listings describing the deviations will be provided. The seizure frequency at baseline and during the course of radiotherapy will be calculated by summing the number of seizures in each period and dividing by the total duration (days), excluding days with no available diary data, and multiplying by 7 to normalize to a weekly rate. The resulting normalized frequencies form the basis for calculating the composite primary endpoint. The point estimate of the rate of progressors and the associated 95% confidence interval will be presented. To test whether the rate of progressions is significantly increased beyond 10%, the one-sided binomial test at a one-sided 2.5% significance level will be applied.

Furthermore, a logistic regression model including baseline seizure including seizure activity at baseline, age (≤59 vs. ≥60 years), gender, glioma grade (grade III vs. IV), recurrent glioma (no vs. yes), IDH1/2 mutation status (mutation vs. wild type), methylation of the MGMT gene promoter (yes vs. no), tumor site (frontal vs. temporal vs. parietal vs. other sites), tumor size (≤ median vs. > median), cortical involvement (yes vs. no) and extent of surgery (no resection/biopsy only vs. subtotal resection vs. gross total resection) will be fitted to identify potentially relevant prognostic factors [14].

Adjusted odds ratios and 95% confidence interval (Wald χ2) will be derived. In addition, each component of the primary composite endpoint will be subjected to a separate statistical analysis using the same methods described above. For exploratory purposes, the analyses described above will also be conducted by focusing on the seizures classified as generalized and generalized/grand mal only.

In order to describe the time profiles of seizure frequencies in more detail, normalized seizure frequencies over time will be calculated within 3-week intervals, namely weeks 1–3 and 4–6 during radiotherapy, and weeks 1–3 (7–9 in total) and 4–6 (10–12 in total) following radiotherapy. These frequencies will be subjected to descriptive statistics as well as graphical presentations by means of box-and whisker diagrams. For exploratory purposes, the Wilcoxon signed-rank test will be applied to compare the two-time windows during the course of radiotherapy; the Friedman-Test will be applied to assess whether there is any difference in seizure frequencies between all of the time intervals during the course of radiotherapy and thereafter.

In addition, the percent change from baseline in seizure frequency will be considered and subjected to descriptive analysis. For further exploratory analysis, the rates of patients experiencing any seizure (yes/no) at baseline and at each time interval will be estimated together with their associated confidence intervals. Subsequent analyses focus on the clinically relevant generalized seizures only.

Patient satisfaction with the seizure diary will be assessed at the end of radiotherapy using a questionnaire and subjected to descriptive analysis. In case of a dissatisfaction rate > 40%, the seizure diary will be considered not suitable for patients with high-grade gliomas. EEG activity typical for epilepsy will be performed during the first and sixth week of radiotherapy, and the sixth week following radiotherapy. EEG activity typical for epilepsy is classified as absent or present. Statistical analysis is focused on data description only. A mean change to baseline (during first week of radiotherapy) by 50% regarding the number of patients with EEG activity typical for epilepsy will be considered clinically relevant.

## Data management and monitoring

Patients can be identified only by the individual patient number, date of birth and gender. A patient list will be kept at the trial center alone. The data will be pseudonymized prior to analyses and handled in accordance with the General Data Protection Regulation. Originals of the key trial documents will be stored at the contributing center (sponsor’s site) for at least 10 years following the final report. The principal investigator will store administrative documents, the patient list, signed informed consent forms, copies of documentation forms and general documentation. Original patient files must be kept for 30 years according to the German regulations for radioprotection. Initiation of the study and on-site monitoring will be performed by the ZKS Lübeck. Regular audits are not planned but may be performed if considered necessary. Since this trial is not related to the German pharmaceutical or medicinal product act, inspections of higher federal authorities are not scheduled. A data monitoring committee is not necessary, because the patients do receive the same treatment for high-grade glioma as outside this trial. The coordinating investigator will work towards comprehensive dissemination of the study findings. Coordinating investigator, biostatistician and staff members involved in the trial will provide a study report. Study results will be published in a peer-reviewed journal and are planned to be presented at scientific meetings. Publications will be coordinated with and supported by a professional biostatistician. For publications the acronym SURF-ROGG will be used. Data analysts and statisticians are blinded.

## Discussion

Primary brain tumors such as gliomas can be associated with seizures. For patients with grade III or IV gliomas, pre-treatment seizure rates of 29–67% and 9–45%, respectively, were reported [3, 15–19]. Moreover, in a study from our group, the prevalence of seizures prior to radiotherapy was 48.8% in patients with grade III gliomas and 21.5% in patients with grade IV gliomas, respectively [14].

Radiotherapy improves long-term seizure outcomes in glioma patients. In a retrospective study of 43 patients with diffuse gliomas (33 patients with a grade II glioma and 10 patients with a grade III glioma), seizure reduction of ≥50% compared to baseline was observed in 31 patients (72%) at 3 months following radiotherapy [20]. Moreover, in a randomized trial comparing early radiotherapy with 54 Gy in 30 fractions to radiotherapy performed only at the time of progression, seizure control at 1 year was better in the early radiotherapy group [21]. However, during the course of radiotherapy, seizures may occur for the first time or the frequency of seizures may increase, because radiotherapy leads to an acute inflammatory reaction, which can be associated with edema and increased intracranial pressure [8, 9]. In 2004, Bhansali et al. reported 11 patients with radiation-induced brain disorders [8]. Four of these patients (36%) had developed generalized seizures. In a review article published in 2007, seizures were reported to be symptoms of both acute radiation encephalopathy and late radiation-related necrosis [9]. However, no study has been available so far that evaluated the effect of irradiation to the brain on seizure activity during the course of radiotherapy for high-grade gliomas. However, these data would be very important in order to improve the monitoring and, if required, the anti-epileptic treatment of these patients. Therefore, the SURF-ROGG trial has been designed that will evaluate the seizure frequency during a course of radiotherapy for high-grade (grade III or IV) gliomas. Patients with low-grade (grade I or II) gliomas will not be included, because the treatment of these tumors and the dose-fractionation of radiotherapy are almost always different from the treatment of high-grade tumors [12].

In this trial, progression of seizure activity is defined as increase of seizure frequency by more than 50%, increase of severity of seizures (i.e. increase of generalized seizures by more than 50%), or initiation or increase of anti-epileptic medication by at least 25%. Evaluation of seizure activity is mainly based on patient-reported outcomes, i.e. on a seizure diary kept by the patients during the period of radiotherapy and up to 6 weeks following radiotherapy. Every day, the patients document the number and type of seizures, as well as the intake of anti-epileptic medication. At the end of radiotherapy, the patients are asked to complete a questionnaire regarding their satisfaction with the seizure diary. In addition to the patient-reported outcomes, EEG activity typical for epilepsy will be evaluated to receive also objective data.

If the SURF-ROGG trial shows an increase in seizure activity during the course of radiotherapy for high-grade gliomas, the findings of this trial may pave the way for a larger prospective trial and will likely contribute to closer patient monitoring and better anti-epileptic treatment.

## Data Availability

Not applicable, because no data have been generated until now. The trial was registered at clinicaltrials.gov (NCT04552756).

## Abbreviations

CTCAE: Common Terminology Criteria for Adverse Events
EEG: Electroencephalography
GCP: Good Clinical Practice
IDH: Isocitrate Dehydrogenase
MGMT: O^6^-Methyl-Guanine-DNA Methyl-Transferase
PCV: Procarbazine, Lomustine and Vincristine
QUANTEC: Quantitative Analyses of Normal Tissue Effects in the Clinic
SURF-ROGG: SeizURe Frequency during a Radiotherapy cOurse for high-Grade Gliomas
TMZ: Temozolomide
VMAT: Volumetric Modulated Arc Therapy
ZKS: Zentrum für Klinische Studien (Centre for Clinical Trials)
